# How are countries responding differently to COVID-19: a systematic review of guidelines on isolation measures

**DOI:** 10.3389/fpubh.2023.1190519

**Published:** 2023-08-30

**Authors:** Guangmei Xie, Li Wang, Jun Zhang

**Affiliations:** ^1^Reproductive Medicine Center, Gansu Maternal and Child Health Care Hospital, Lanzhou, Gansu, China; ^2^Reproductive Medicine Center, Gansu Provincial Central Hospital, Lanzhou, Gansu, China; ^3^School of Nursing, Gansu University of Chinese Medicine, Lanzhou, Gansu, China

**Keywords:** COVID-19, isolation, guidance, guidelines, systematic review

## Abstract

**Introduction:**

Isolation strategies have been implemented in numerous countries worldwide during the ongoing community transmission of severe acute respiratory syndrome coronavirus 2 (SARS-CoV-2). However, various countries and organizations have implemented their isolation measures at varying intensities, even during the same period. Therefore, we systematically reviewed the key information contained in currently available guidelines regarding the isolation of the general population, aiming to better identify the heterogeneity of the current isolation strategies.

**Methods:**

We conducted searches in four evidence-based medicine (EBM) databases and five guideline websites to identify guidelines, guidance, protocols, and policy documents published by authoritative advisory bodies or healthcare organizations, which provided information on the implementation of isolation for general populations with COVID-19. One author extracted data using a standardized data extraction checklist, and a second author double-checked all extractions for completeness and correctness. Discrepancies were resolved through discussion. The information extracted from the included articles was summarized both narratively and using tables.

**Results:**

We included 15 articles that provided information on isolation measures recommended by nine different countries and organizations. The included articles consistently recommended isolating individuals with a positive COVID-19 test, regardless of the presence of symptoms. However, there were variations in the duration of isolation, and substantial differences also existed in the criteria for ending the isolation of COVID-19 patients.

**Conclusion:**

Different countries and organizations have substantial differences in their isolation policies. This reminds us that scientifically sound guidelines on isolation that balance the risk of prematurely ending isolation with the burden of prolonged isolation are a crucial topic of discussion when faced with a pandemic.

## 1. Introduction

It has been nearly 4 years since the first coronavirus disease 2019 (COVID-19) case was reported in Wuhan, China (1). According to the WHO dashboard, millions of COVID-19 cases are diagnosed worldwide every week (2). Among these newly diagnosed cases, many are laboratory-confirmed with no symptoms (3). For patients who develop symptoms, the majority experience mild or moderate disease (4–6). This situation is mainly attributed to vaccine-induced and infection-induced immunity, as well as the emergence of new variants (7–10).

While severe COVID-19, hospitalization, and death decreased in infected individuals, the Omicron variant showcased increased transmissibility as its main characteristic (11–16). The Omicron virus has caused waves of infections worldwide as the Omicron variant of concern (VOC), suggesting that the pandemic may persist for a longer duration. Although most patients recovered either spontaneously or with acute-phase management, the global healthcare and economies suffered a significant consequence. Moreover, a proportion of individuals infected with severe acute respiratory syndrome coronavirus 2 (SARS-CoV-2) experienced long-term COVID-19 complications, regardless of the initial severity of infection, exhibiting a diverse range of symptoms (17). Guidelines for managing long-term COVID-19 recommend that the prevention of SARS-CoV-2 is the most effective approach to preventing the post-COVID state (18, 19). Consequently, it remains a global priority to prevent COVID-19 transmission and the subsequent impact on associated illnesses and deaths.

Early in the pandemic, isolation was pivotal in controlling the outbreak (20, 21). Similarly, in the context of the ongoing community transmission of SARS-CoV-2, isolation strategies continue to be crucial and effective non-pharmaceutical interventions that have been implemented in many countries worldwide. However, it is important to note that different countries and organizations have implemented these strategies with varying degrees of intensity.

Therefore, in order to better identify inconsistent isolation measures, we have summarized the key information regarding the isolation of the general population as outlined in current guidelines or guidance documents.

## 2. Methods

This systematic review followed the Preferred Reporting Items for Systematic Reviews and Meta-Analyses (PRISMA) guidelines (22).

### 2.1. Inclusion criteria

We included guidelines, policy statements, protocol documents, and interim guidance documents published by authoritative advising bodies or healthcare organizations, which provide information on implementing isolation for general populations with COVID-19. We identified the latest version of each eligible article. For feasibility reasons, articles had to be published in English or Chinese.

### 2.2. Information sources

An experienced team member specializing in systematic reviews conducted electronic searches on four evidence-based medicine (EBM) databases, namely Clinicalkey, UpToDate, Best Practices, and DynaMed Plus. In addition, we searched other sources of guidelines, including the Guidelines International Network (GIN), the National Institute for Health and Clinical Excellence (NICE), the Scottish Intercollegiate Guidelines Network (SIGN), the Chinese Medlive Guidelines Network, and the website of WHO. Articles were identified using the keywords “COVID-19” and “novel coronavirus pneumonia”. Furthermore, the reference lists of topic-related reviews were hand-searched to supplement the electronic database searches. The initial search was performed in September 2022, and an update was conducted in December 2022.

### 2.3. Selection of studies

An experienced review author screened the literature searches based on the title or descriptors, excluding the articles that clearly did not meet the inclusion criteria of this review. The full texts of all included titles were retrieved. Two review authors independently screened all full-text articles to assess their eligibility for inclusion. Any disagreements were resolved through discussion or by seeking an independent third opinion.

### 2.4. Quality assessment

Producing COVID-19-related guidelines may not follow the standards for developing guidelines compared with usual times because COVID-19 is an urgent global health threat that needs prompt responses. Additionally, this review aimed to provide a comprehensive overview of the isolation measures adopted by different countries. Our intention was not to select the optimal isolation policy. Therefore, the methodological and reporting quality of included articles was not assessed.

### 2.5. Data extraction

One experienced review author used a standardized data extraction checklist to extract data from the included articles. A second reviewer double-checked all extractions to ensure completeness and correctness. Any disagreements were documented and resolved through discussion, if necessary. The data items included (1) characteristics of articles (e.g., name, source, and date); (2) population to be isolated—the definitions of different populations (e.g., asymptomatic, mild, and moderate cases) were defined as reported by the authors; (3) setting of isolation (e.g., personal residence, community facility, and health facility); (4) when to discontinue isolation; and (5) criteria for releasing individuals from isolation.

### 2.6. Data synthesis

We presented the information extracted from the included articles in narrative format and summary tables.

## 3. Results

### 3.1. Study selection and characteristics of included articles

**Figure 1:**
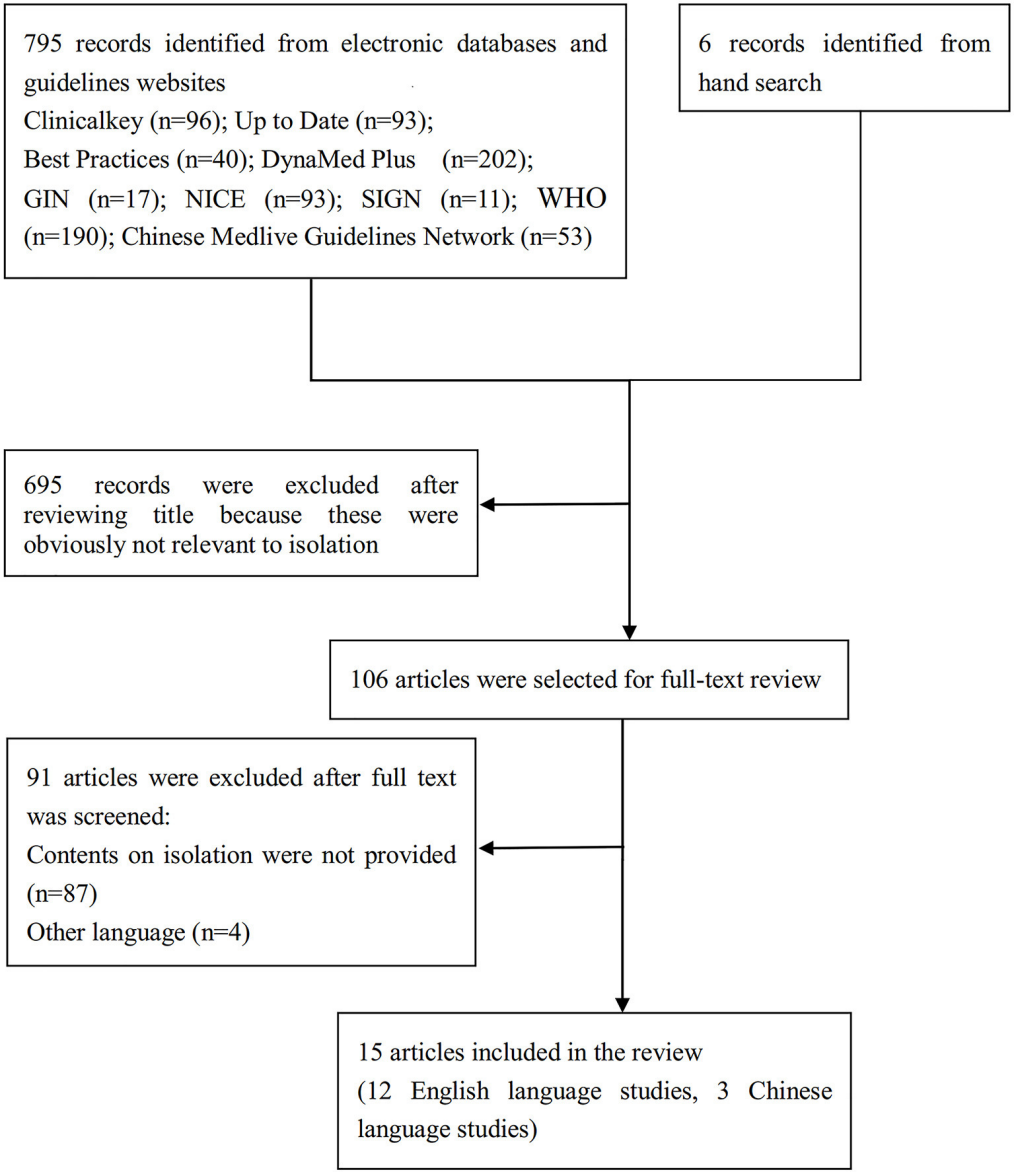
PRISMA flow diagram showing article selection.

We screened 801 titles retrieved from our electronic search of databases, guideline websites, and hand search. Out of the 106 full texts that were retrieved for further assessment, 15 relevant articles (23–37) fulfilled our eligibility criteria. We also examined the references cited in published relevant review articles until no additional articles were found. Figure 1 presents a flow diagram illustrating the study selection process, and detailed characteristics of the included articles are presented in Table 1. The included articles (23–37) provided information on isolation measures recommended by nine different countries and organizations, namely Australia, Canada, China, India, the European Union (EU), Saudi Arabia, the United Kingdom (UK), the United States (US), and the World Health Organization (WHO). Most of the articles were interim guidance, while others included guidelines, protocols, and statements. For convenience reasons, we will refer to all these articles as “guidance” henceforth.

**Table 1:** Characteristics of included guidance by country or organization.

**Title**	**Development institute**	**Country or organization**	**Last available update date**
Coronavirus Disease 2019 (COVID-19) CDNA National Guidelines for Public Health Units (23)	Communicable Disease Network Australia (CDNA)	Australia	9 September 2022
AHPPC Statement—Reduced Isolation Period for COVID-19 Cases (24)	The Australian Health Protection Principal Committee (AHPPC)	Australia	8 September 2022
Public Health Management of Cases and Contacts associated with COVID-19 (25)	The Public Health Agency of Canada (PHAC)	Canada	24 December 2021
Guidance on Further Optimizing the Measures for the Prevention and Control of COVID-19 (26)	State Council	China	7 December 2022
Protocol for Prevention and Control of COVID-19 (Version 9) (27)	National Health Commission	China	27 June 2022
Protocol for Diagnosis and Treatment of COVID-19 (Version 9) (28)	National Health Commission	China	15 March 2022
Guidance on Ending the Isolation Period for People with COVID-19, Third Update (29)	European Centre for Disease Prevention and Control (ECDC)	European Union (EU)	28 January 2022
Revised Discharge Policy for COVID-19 (30)	Ministry of Health & Family Welfare (MHFW)	India	9 January 2022
Revised Guidelines for Home Isolation of Mild/Asymptomatic COVID-19 Cases (31)	MHFW	India	5 January 2022
Clinical Management Protocol for COVID-19 (in Adults) Version 6 (32)	MHFW	India	24 May 2021
Coronavirus Disease COVID-19 GUIDELINES (33)	Ministry of Health	Saudi Arabia	4 January 2022
Guidance for People with Symptoms of a Respiratory Infection Including COVID-19, or a Positive Test Result for COVID-19 (34)	Health Security Agency	UK	1 April 2022
Ending Isolation and Precautions for People With COVID-19: Interim Guidance (35)	Center for Disease Control and Prevention (CDC)	US	31 August 2022
CDC Streamlines COVID-19 Guidance to Help the Public Better Protect Themselves and Understand Their Risk (36)	CDC	US	11 August 2022
Clinical Management of COVID-19: Living Guideline (37)	WHO	WHO	15 September 2022

### 3.2. People who need isolation

All included guidance recommended isolating individuals with a positive COVID-19 test result, regardless of whether they have symptoms or not.

### 3.3. Locations of isolation

Most of the included guidance recommended home isolation for patients with asymptomatic or mild COVID-19, including moderate cases. However, WHO highlighted that the decision to monitor a symptomatic case in a health facility, community facility, or home should depend on the clinical presentation, need for supportive care, risk factors, and conditions at the private residence (37). Patients with one or more risk factors for rapid COVID-19 deterioration, severe disease, and increased mortality should preferably be referred to a health facility for monitoring and treatment. It is worth mentioning that several countries and regions, such as China (26, 27), India (31, 32), Saudi Arabia (33), and the EU (29), have fully or partly acknowledged these factors in their policies.

### 3.4. Duration of isolation

As presented in Table 2, the recommendations for isolation duration were influenced by factors such as the severity of the disease, the vaccination status of the person exposed, and their immunosuppression status.

**Table 2:** Criteria for ending isolation in the included guidance.

**Countries**	**Asymptomatic cases**	**Symptomatic cases**
Australia	Five days after their first positive test and the case remained asymptomatic.	Cases can be released from isolation 5 days after their first positive test if they meet the following criteria: substantial resolution of their acute respiratory symptoms; no fever for 24 h without the use of fever-reducing medications.
Canada	At least 10 days have passed since the date their positive specimen was collected, and the case remained asymptomatic.	Isolation can be discontinued at least 10 days after the first symptom onset if the ever has resolved and clinical symptoms have improved (absence of anosmia or fatigue/tiredness should not be required; absence of cough should also not be required for those known to have chronic cough or for those who are experiencing reactive airways post infection).
China	The case remained asymptomatic seven days after the sample for the positive test was taken and two consecutive negative RT-PCR tests on days 6 and 7.	Patients with mild illness: 7 days after the sample for the positive test was taken AND two negative RT-PCR tests from respiratory specimens on days 6 and 7. • Patients with moderate to critical illness: cases can be released from hospital isolation when they meet the following criteria: two negative PCR tests at least 24 h apart; substantial resolution of their respiratory symptoms; no fever for 3 successive days; and pulmonary imaging showed significant improvement in acute exudative lesions. • Home quarantine can be exited if asymptomatic cases test negative on days 1, 4, and 7.
European Union	Not fully vaccinated: 10 days after the sample for the positive test was taken. • Fully vaccinated: 6 days after the sample for the positive test was taken and one negative RADT or RT-PCR test from respiratory specimens on day 6. • Not fully vaccinated residents or staff of closed vulnerable population settings: 20 days after the sample for the positive test was taken. • Fully vaccinated residents or staff of closed vulnerable population settings: 10 days after the date of the sample collection for their diagnostic test and one negative RADT or RT-PCR test from respiratory specimens on day 10. • Not vaccinated or not fully vaccinated, mild or moderate COVID-19 case with essential work: 10 days isolation after the onset of symptoms. • Fully vaccinated and mild or moderate COVID-19 cases with essential work: 6 days of isolation after the date of the sample collection for their diagnostic test and a negative RADT or RT-PCR test from respiratory specimens on day 6 OR the patient above can be released from isolation when they have two consecutive negative SARS-CoV-2 RADT or RT-PCR tests from respiratory specimens with a minimum 24-h interval.	Mild/moderate COVID-19: - Not fully vaccinated: the resolution of fever, if present, for at least 24 h and clinical improvement of symptoms other than fever; 10 days after the onset of symptoms. - Fully vaccinated: the resolution of fever, if present, for at least 24 h and clinical improvement of symptoms other than fever; 6 days after the onset of symptoms; one negative RADT or RT-PCR test from respiratory specimens on day 6 or later. • Severe COVID-19: the resolution of fever for at least 24 h and clinical improvement of symptoms other than fever; minimum 14 and up to 20 days after the onset of symptoms. • Immunocompromised patient: the resolution of fever, if present, for at least 24 h and clinical improvement of symptoms other than fever; or 20 days after the onset of symptoms. • Not fully vaccinated residents or staff of closed vulnerable population settings: the resolution of fever, if present, for at least 24 h and clinical improvement of symptoms other than fever; 20 days after the onset of symptoms. • Fully vaccinated residents or staff of closed vulnerable population settings: the resolution of fever, if present, for at least 24 h and clinical improvement of symptoms other than fever; 10 days after the onset of symptoms; one negative RADT or RT-PCR test from respiratory specimens on day 10. • Not vaccinated or not fully vaccinated and mild or moderate COVID-19 case with essential work: the resolution of fever for 24 h and clinical improvement of symptoms; 10 days isolation after the onset of symptoms. • Fully vaccinated and mild or moderate COVID-19 cases with essential work: the resolution of fever for 24 h and clinical improvement of symptoms; 6 days after the onset of symptoms; a negative RADT or RT-PCR test from respiratory specimens on day 6 OR the patient above can be released from isolation after two consecutive negative SARS-CoV-2 RADT or RT-PCR tests from respiratory specimens 24 h apart.
India	At least 7 days have passed from testing positive, and the case remained asymptomatic.	Mild cases: at least 7 days have passed from testing positive and no fever for three successive days. • Moderate cases: symptoms are resolved, and the patient maintains saturation above 93% for three successive days without oxygen support and stable comorbidities. • Severe cases: discharge criteria will be based on clinical recovery at the discretion of the treating medical officer.
Saudi Arabia	Unimmunized: 10 days have passed since the date of collection of the respiratory sample with the first positive RT-PCR result. • Immunized: 7 days have passed since the date of collection of the respiratory sample with the first positive RT-PCR result.	Immunized: 7 days after the onset of symptoms and resolution of fever for at least 24 h without antipyretics. • Unimmunized: - Mild confirmed cases: 10 days after the onset of symptoms and resolution of fever for at least 3 days, and clinical improvement of other symptoms. - Severe infection: at least 10 days have passed since the onset of symptoms, no recorded fever in the last 3 days without using antipyretics, and improvement of other symptoms (Cough, SOB, and GI symptoms). - Severe infection with immunocompromised and critical cases (ICU admitted patients): at least 21 days after symptom onset and resolution of fever for at least 3 days, and clinical improvement of symptoms other than fever (Cough, SOB, and GI symptoms) OR at least 3 days have passed since recovery [resolution of fever without using fever-reducing medication and symptom improvement (Cough, SOB, and GI symptoms)] and followed by two negative respiratory samples more than 24 h apart.
UK	Children and young people aged 18 years and younger: if they feel well and without fever 3 days after their first positive test. • Adults: at least 5 days after their first positive test, until they feel well-enough to resume normal activities and no longer have a fever if they had one.	Children and young people aged 18 years and younger: 3 days after their first positive test if they feel well and do not have a fever. • Adults: at least 5 days from their first positive test until they feel well-enough to resume normal activities and no longer have a fever if they had one.
US	Isolation can be discontinued at least 5 days after the first positive viral test.	People with mild COVID-19: isolation can be discontinued at least 5 days after symptom onset if fever has resolved for at least 24 h (without taking antipyretics) and other symptoms are improving. A high-quality mask should be worn around others at home and in public through day 10. A test-based strategy may be used to remove a mask sooner. • People with moderate COVID-19: isolation and precautions can be discontinued 10 days after symptom onset. • People with severe COVID-19: isolation can be discontinued at least 10 days after symptom onset. • For those with severe illness (e.g., requiring hospitalization, intensive care, or ventilation support), isolation can be discontinued at least 20 days after symptom onset and after resolution of fever for at least 24 h (without taking antipyretics) and improvement of other symptoms. Serial testing before ending isolation can be considered in consultation with infectious disease experts. • People with moderately or severely immunocompromised (regardless of COVID-19 symptoms or severity): results are negative from at least two consecutive respiratory specimens collected more than 24 h apart (total of two negative specimens) tested using an antigen test or nucleic acid amplification test. If a moderately or severely immunocompromised patient with COVID-19 was symptomatic, there should be resolution of fever for at least 24 h (without taking antipyretics) and improvement of other symptoms.
WHO	If no testing is available, isolation can be discontinued at least 10 days after a positive test for SARS-CoV-2. • If testing is available, two negative PCR tests at least 24 h apart can be used.	If no testing is available, isolation can be discontinued at least 10 days after symptom onset, plus at least three additional days without symptoms (without fever and respiratory symptoms). • If testing is available, two negative PCR tests at least 24 h apart can be used.

Generally, in most included guidance, the isolation period for asymptomatic and mild cases was reduced to 5–7 days. However, individuals with more severe disease and weakened immunity were advised to have a longer isolation duration. Of note, we found revealed significant variation in these recommendations across different countries for patients with similar conditions. For example, an 18-year-old asymptomatic patient would be required to have an isolation period of only 3 days in the UK (34), whereas, in Saudi Arabia, the recommended isolation period would be 10 days (33).

### 3.5. Criteria for releasing individuals from isolation

As shown in Table 2, asymptomatic cases could be released from isolation once they complete the necessary duration of isolation and remain asymptomatic, except for guidance from China and the EU (26, 29). China and the EU recommended implementing an antigen detection rapid diagnostic test (Ag-RAT) or reverse transcription polymerase chain reaction test (RT-PCR) to confirm the absence of contagious virus when ending isolation.

For symptomatic cases, in addition to the recommended duration of isolation, the improvement of symptoms was the most common requirement for ending isolation, usually including the resolution of fever without using antipyretics and substantial improvement in respiratory symptoms. Moreover, the guidance from India and China also includes some physiological indicators (28, 30). Nevertheless, there is disagreement among different countries regarding the necessity of testing. While some countries did not recommend repeat testing for SARS-CoV-2 as the basis for discontinuing isolation, WHO still suggested that countries may continue using testing as part of the release criteria (37), and several countries have adopted this suggestion.

## 4. Discussion

This systematic review provides a comprehensive summary of available guidance documents that report information on isolation measures for the general population with COVID-19. The results of the review indicate significant heterogeneity in isolation policies across different counties and regions, particularly pertaining to the duration of isolation and the criteria for ending isolation. Moreover, this review offers some insights into relevant implementation strategies.

### 4.1. Who needs isolation?

As we know, controlling the spread of infectious sources is crucial for mitigating diseases. In the case of the current SARS-CoV-2, evidence has demonstrated that individuals who are asymptomatic at the time of testing, as well as those who are pre-symptomatic, can shed replicating virus to their close contacts (38, 39). Additionally, studies have shown that the viral load in the upper respiratory tract and the probability of detecting viable viruses are comparable between asymptomatic individuals and those with symptomatic SARS-CoV-2 infection (40, 41). This indicates that asymptomatic patients can serve as a source of SARS-CoV-2 transmission. Consequently, all the guidance analyzed in this review recommended isolating COVID-19 patients, regardless of whether they exhibit symptoms.

### 4.2. Where to isolate?

During the period dominated by the Omicron variant, home isolation has been widely adopted by many countries in comparison to centralized isolation. The preference for home isolation can be explained by the fact that isolating in a community or health facility incurs high economic, societal, and psychological costs. In addition, most cases do not require hospital-level care to recover. Of note, a high household secondary attack rate has been reported in many countries (42–45), including 31.8% in Japan (43), 50% in Korea (44), 52.7% in the US (45), and 80.9% in Spain (42). These elevated attack rates may indicate a low level of compliance with home isolation measures among index-case patients and their households. This suggests that the effectiveness of isolation would significantly decrease without adequate implementation of infection prevention and control measures recommended for isolated individuals and their households.

In addition to that, the mental health of individuals who are isolated at home should not be overlooked. While psychological issues are significantly higher in individuals in centralized isolation than in those isolated at home, it does not mean that people who isolate at home will not experience psychological problems. Many studies have indicated that prolonged periods of home isolation can still lead to feelings of loneliness, anxiety, depression, and other negative emotions (46–48). Therefore, when implementing home isolation measures, it is important to consider the negative impact of home isolation and take steps to address and support people's mental wellbeing by providing resources such as psychological counseling and support services to alleviate the psychological distress they may be facing.

### 4.3. How long to isolate?

Scientifically sound guidelines on isolation that strike a balance between the risk of prematurely ending isolation and the burden of prolonged isolation are crucial to discuss. The ideal duration of isolation after infection should be determined based on the transmissibility of the current VOC. Regarding individuals infected with the SARS-CoV-2 Omicron variant, Jang et al. analyzed the duration of the infectious stage using viral culture of respiratory samples. The result found that all samples taken 9 days after symptom onset showed negative viral cultures (49). Similarly, a comparative analysis of the transmissibility period reported that Omicron infections featured a mean duration of 9.87 days inferred from the viral load (50). While viral load or cell culture infectivity cannot be directly translated to transmission probability, they are commonly used as proxies to estimate infectiousness and hence transmission.

We learned from the guidance included that many countries have shortened the recommended duration of isolation from 10 to 5 days. However, this recommendation was received with skepticism, as many people think that there was insufficient fully elaborated scientific evidence to support this decision. Recent data from the US indicate that 35% of symptomatic non-severe individuals infected with the Omicron variant, despite having received a booster vaccine, continued to shed culture-able virus more than 5 days after the onset of symptoms or an initial positive test (51). Similarly, a study conducted in Turkey showed that among symptomatic non-severe SARS-CoV-2 Omicron variant infected patients, 83% shed infectious viral particles on day 5, 52% on day 7, 13.5% on day 10, and 8.5% on day 14 (52). This suggests that some cases may still be infectious at the end of the recommended isolation period.

Although it is advised for these individuals to wear a high-quality mask when around others at home and in public after the cessation of isolation on day 5 (35), it is impossible to follow up on the cases on whether they maintain isolation precautions while working. Therefore, the potential increase in virus transmission due to infectious residual viral load related to non-adherence to the recommended mitigation measures is a dramatic challenge to the global response to COVID-19.

Additionally, it is worth considering whether isolation measures should be relaxed for vaccinated individuals. Preliminary evidence suggests that the duration of viral shedding may be shorter and clearance more rapid in vaccinated patients who are infected with recently emerged VOCs (53). As a result, guidance has recommended a shorter isolation period for vaccinated individuals. However, recently published studies have indicated conflicting results regarding the viral infection dynamics of the Omicron variant. One study by Selvavinayagam et al. reported that the viral load was generally lower among vaccinated individuals than in non-vaccinated infected individuals (54). Conversely, Puhach et al. found that reduced infectious viral load was observed only in boosted individuals, not in fully vaccinated individuals, when compared to unvaccinated individuals (55). However, a study published in the New England Journal of Medicine found no significant differences in the median duration of viral shedding among unvaccinated participants, participants who were vaccinated but not boosted, and participants who were both vaccinated and boosted (51). Possible reasons for this conflicting result may include characteristics of the study population, type of vaccine received, history of SARS-CoV-2 infection, underlying comorbidities, and type of VOC. Of course, conducting a systematic review that synthesizes multiple studies regarding this topic is an optimal way to address the inconsistency.

### 4.4. How to release from isolation?

The most current guidance recommends a symptom-based strategy as an additional criterion for ending the isolation of symptomatic COVID-19 patients, after a necessary time interval has elapsed since the onset of symptoms. This approach avoids the use of SARS-CoV-2 testing, particularly for immunocompetent patients with mild-to-moderate COVID-19. Nevertheless, recent evidence suggests that this policy may not be reliable. According to Jang et al. there is a weak correlation between the duration of fever and the time taken for viral culture conversion in patients infected with the Omicron variant (49). Keske et al. reported that among SARS-CoV-2-confirmed patients who stated that their symptoms had resolved, the rate of detected viral shedding was 58% on day 7, 11% on day 10, and 5% on day 14 (52). These findings suggest that the resolution of symptoms does not guarantee the absence of viral shedding.

Furthermore, several studies have compared different criteria for determining when to end isolation using mathematical models. The findings of these studies indicate that implementing testing protocols for isolated individuals can help minimize unnecessary isolation while still effectively controlling the risk of further transmission (56–58). Additionally, some studies have evaluated the use of antigen tests to guide the end of isolation and consistently concluded that using these tests may reduce redundant isolations or prevent forward transmission (58–60). However, it is worth noting that many sets of guidance do not advocate for repeating laboratory testing as the sole basis for discontinuing isolation, primarily due to resources and cost considerations. Nonetheless, laboratory testing does provide a more accurate assessment of ongoing risk. Thus, when preparing for future pandemics, such as the COVID-19 outbreak, governments should prioritize the development of effective and cost-efficient testing methods.

### 4.5. Limitations

There are several limitations in retrieving and reviewing the guidance. First, our search was limited to the EBM databases and guideline websites, potentially missing out on guidance published by other countries and the latest version of the included guidance. Second, we only included guidance documents in English or Chinese, potentially overlooking those published in other languages. Third, we utilized descriptive analysis alone to address the research question, without incorporating additional evidence from data analysis. Finally, our review was not registered in PROSPERO as it did not meet the inclusion criteria. These limitations may potentially impact the reliability of the results. However, we believe that the included sets of guidance are representative, indicating that the contentiousness on isolation reflected by the included guidance may likely exist in other guidance as well.

## 5. Conclusion

Different countries and organizations have substantial differences in their isolation policies for COVID-19. The findings of this study remind us that, in dealing with similar pandemics in the future, decision-makers should take into account the negative impacts of isolation on individuals and society while effectively curbing virus transmission. Additionally, it is imperative to prioritize the development of cost-effective laboratory tests that can inform scientifically accurate isolation policies, thereby avoiding the risk of prematurely ending isolation and the burden of prolonged isolation.

## Data availability statement

The original contributions presented in the study are included in the article/supplementary material, further inquiries can be directed to the corresponding author.

## Author contributions

JZ and GX participated in the conception and design of the study. GX and LW contributed to data collection and analysis. All authors drafted and critically reviewed this manuscript and approved the final version.
